# EEG recordings during visuo‐attentive task reduce sex bias in Alzheimer's disease diagnosis

**DOI:** 10.1002/trc2.70245

**Published:** 2026-04-06

**Authors:** Lorenzo Gaetano Amato, Michael Lassi, Antonello Grippo, Valentina Moschini, Giulia Giacomucci, Sonia Padiglioni, Carmen Morinelli, Benedetta Nacmias, Alberto Mazzoni, Valentina Bessi, Alberto Arturo Vergani

**Affiliations:** ^1^ The BioRobotics Institute Sant'Anna School of Advanced Studies Pontedera Italy; ^2^ Department of Excellence in Robotics and AI Sant'Anna School of Advanced Studies Pisa Italy; ^3^ IRCCS Fondazione Don Carlo Gnocchi Florence Italy; ^4^ Department of Neuroscience, Psychology, Drug Research and Child Health Careggi University Hospital Florence Italy; ^5^ Neurology Unit, Dipartimento Neuromuscoloscheletrico E Degli Organi Di Senso Careggi University Hospital Florence Italy; ^6^ Regional Referral Centre for Relational Criticalities ‐ Tuscany Region Florence Italy; ^7^ Department of Brain and Behavioral Sciences University of Pavia Pavia Italy

**Keywords:** electroencephalography, event‐related potentials, machine learning, sex differences, subjective cognitive decline

## Abstract

**INTRODUCTION:**

Pathological cognitive decline affects roughly 15% of older adults, presenting relevant sex differences, being a stronger predictor of Alzheimer's disease in females. Yet, sex‐specific neural markers that support a balanced early diagnosis remain limited. Event‐related potentials (ERPs) derived from task‐based electroencephalograph (EEG) offer a non‐invasive window into cognitive processing, but their sex‐specific diagnostic value in subjective cognitive decline (SCD) has not been explored.

**METHODS:**

We recorded EEG during a visuo‐attentive task in elders with SCD (*n* = 119) and age‐matched healthy controls (*n* = 19) to extract ERP components reflecting stimulus encoding and decision processes. We investigated sex‐specific associations between ERP features and task performance and trained separate machine learning models for SCD diagnosis in males and females. Diagnostic models were constructed using either clinical features alone or a combination of clinical and ERP features to quantify the added value of ERPs over standard assessments.

**RESULTS:**

ERP analyses revealed distinct sex‐specific associations between neural responses and behavioral performance, suggesting partially divergent neurocognitive mechanisms underlying attentional processing in aging males and females. Diagnostic models based solely on clinical data produced significantly unbalanced performance between sexes (area under the curve [AUC]: 0.75 males, AUC: 0.63 females; *p* < 0.00001). When ERP features were incorporated, classification accuracy improved in both groups and the sex imbalance was eliminated (AUC: 0.77 males, AUC: 0.75 females; *p* = 0.31). ERP features consistently demonstrated higher sensitivity to subtle cognitive alterations, particularly in females.

**DISCUSSION:**

Task‐related ERPs provide complementary, sex‐specific neural information that mitigates diagnostic disparity arising from clinical assessments alone. Incorporating ERP features supports a more equitable early identification of cognitive decline and may improve screening strategies for populations usually under‐recognized in preclinical Alzheimer's disease pathways.

**TRIAL REGISTRATION:**

ClinicalTrials.gov identifier: NCT05569083

**Highlights:**

- Task‐based electroencephalograph (EEG) reveals sex‐specific neural mechanisms in early Alzheimer's disease (AD) stages.
- Event‐related potentials (ERP) features improve subjective cognitive decline (SCD) diagnosis and eliminate sex bias in classification.
- Combining cognitive data with task‐EEG enhances overall diagnostic accuracy.
- Visuo‐attentive EEG offers a non‐invasive, unbiased tool for early AD detection.

## 1 BACKGROUND

During the aging process, a percentage between 10% and 15% of individuals aged >45 years experience a condition known as subjective cognitive decline (SCD),^1^, ^2^, ^3^, ^4^ characterized by abnormal deterioration of cognitive functions.^5^ SCD is recognized as a preclinical stage of dementia, with high transition rates toward more severe forms^1^, ^2^ and is currently acknowledged as a prodromic form of Alzheimer's disease^4^ (AD). For its high social and economic impact, the characterization of the SCD condition and the prediction of transition toward more severe forms of dementia has become a crucial problem in our society.^5^

Notably, cognitive decline and AD dementia present a relevant sex‐specificity, being more prevalent in females than in males in older inividuals.^6^ SCD is a stronger indicator of further dementia progression in females when compared to males.^7^, ^8^ Moreover, dementia incidence appears to be higher for females,^9^, ^10^, ^11^ who also present higher susceptibility to neurodegeneration^11^, ^12^, ^13^ and difficulties in proper identification of the condition.^14^ This higher vulnerability in females may be influenced by social factors,^6^, ^10^ in addition to biological inter‐sex differences. The existence of sex‐specific mechanisms in dementia progression is currently debated,^15^ and the identification of sex‐specific biomarkers of clinical cognitive decline and dementia remains scarcely addressed,^16^ particularly in preclinical stages such as SCD.

The use of electroencephalography (EEG) to characterize early stages of AD and cognitive decline is consolidated in both clinical practice and research.^17^, ^18^, ^19^, ^20^ Compared to conventional neuroimaging techniques, the EEG offers several advantages, including wider availability, lower costs,^21^ and high potential in capturing functional brain changes associated with the earliest stages of cognitive decline.^18^, ^22^ EEG recordings are often acquired during cognitive task execution to identify the neural correlates of cognitive dysfunctions in a vast array of tasks (e.g., the Stroop task,^23^ item list recall,^24^ tone discrimination,^25^ and visuo‐attentive tasks).^26^, ^27^ These deficits reflect underlying disruptions in neural networks critical for information processing, encoding, and decision‐making.^27^, ^28^ However, the sex‐specific differences in cognitive performance during task execution have received limited attention, especially along the AD spectrum.

**FIGURE 1:**
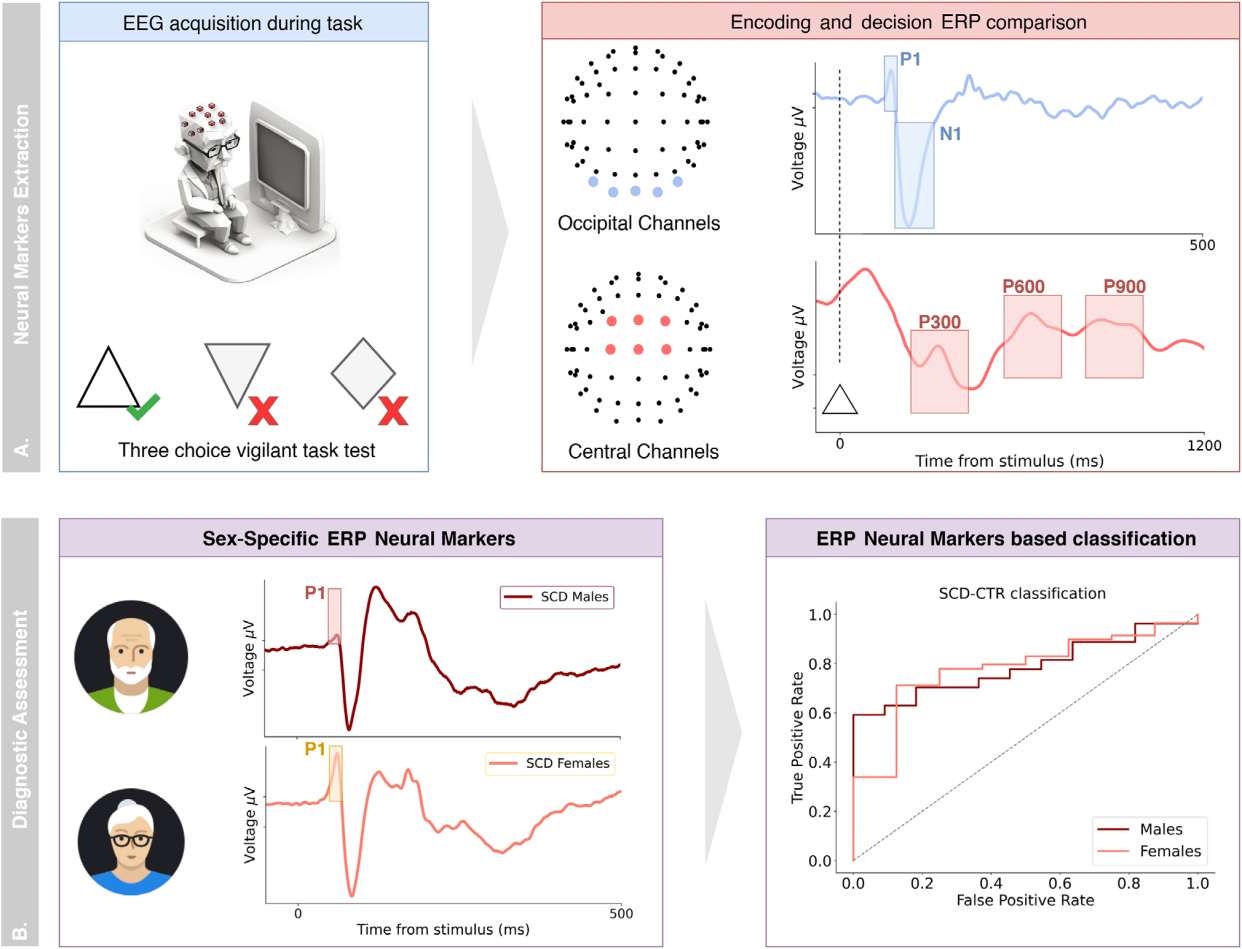
Using ERP features to uncover sex‐specific mechanisms underlying the SCD condition and reducing sex‐bias in early diagnosis. (A) Participants in healthy (CTR) and SCD conditions were enrolled. EEG recordings were then collected during cognitive task execution, and results were analyzed to extract neural markers (ERP features) from occipital and central channels, reflecting encoding (P1 and N1 components from occipital channels) and decision (P300, P600 and P900 components from central channels) processes. (B) Sex‐specific analysis of ERP features was conducted. Extracted neural markers were then validated by using them as classifying features to support a sex‐specific CTR versus SCD classification. CTR, control; EEG, electroencephalography; ERP, event‐related potential; SCD, subjective cognitive decline.

In this study, we analyzed EEG signals acquired during the execution of a three‐choice vigilant task (Figure 1A) in SCD participants (*n* = 119) and age‐matched healthy controls (CTR, *n* = 19). EEG data were processed to extract event‐related potential (ERP) components: EEG waveforms associated with encoding and decision‐making processes.^27^, ^28^ ERP component analysis revealed sex‐specific mechanisms associating neural activity with task performance and provided an equitable SCD‐CTR classification, significantly reducing the sex bias observed in the classification made without EEG data.

## 2 METHODS

### 2.1 Participants recruitment and task architecture

Extensive information about participant recruitment, inclusion and exclusion criteria, and the neuropsychological test battery can be consulted in Mazzeo et al.^29^ and on the clinical trial site of the PREVIEW project, and are summarized in Supporting Information.

### 2.2 EEG recordings and pre‐processing

The EEG recordings were conducted with subjects seated in a comfortable position during the execution of the 3‐CTV task. Signals were collected using the 64‐channel Galileo‐NT system (E.B. Neuro S.p.A.), with sensor topography following the extended 10/20 system. Unipolar signals were recorded at a sampling rate of 512 Hz, and electrode impedances, monitored throughout EEG acquisition, were maintained in the 7–10 kΩ range. The EEG pre‐processing pipeline was implemented using MATLAB with the commercially available EEGLAB Toolbox.^30^ Pre‐processing followed the steps delineated by Vergani et al.,^27^ and is extensively discussed in the Supporting Information, alongside the procedure adopted for ERP extraction and quality control of signal preprocessing (Table S1).

### 2.3 Statistical analysis and software

Binary comparisons were conducted using the Mann–Whitney U‐test, while Kruskal–Wallis H‐test was used for three‐group comparisons, using Mann–Whitney with Bonferroni correction for pairwise comparisons. Statistical tests for categorical quantities such as biological sex were conducted with the *χ* ^2^ test.

Correlation analyses were employed to detect relationships between variables, notably between biological quantities such as age and cognitive metrics. To compare correlations in different groups, we used the Fisher *Z*‐test.

Th machine learning pipeline is extensively discussed in the Supporting Information. Mann–Whitney U‐test was used to compare classifiers’ performances, computing effect sizes with Cohen's *d*. All machine‐learning and data analysis pipelines were implemented in Python, using standard packages such as SciPy, NumPy, SciKit Learn, and Pandas. Standardized effect sizes were computed in combination with the Bayes factor (BF), whose values are adjusted for the class imbalance between groups (see Supporting Information).

**RESEARCH IN CONTEXT**

1. **Systematic review**: We reviewed recent literature on sex differences in cognitive decline and Alzheimer's disease (AD). Although sex disparities in cognitive trajectories are well documented, existing diagnostic frameworks rarely address sex‐specific neural mechanisms. Prior electroencephalograph (EEG) research has primarily relied on resting‐state data, providing limited insight into task‐evoked neural responses relevant to cognitive function.
2. **Interpretation**: Our findings demonstrate that EEG responses during visuo‐attentive tasks capture sex‐specific neural signatures that improve early‐stage AD diagnosis. By integrating event‐related potentials (ERP) with clinical data, we achieved balanced diagnostic performance between males and females, overcoming sex bias inherent in clinical scores alone.
3. **Future directions**: Future research should investigate whether task‐evoked EEG markers can generalize to larger and more diverse populations, extend to preclinical AD stages and elucidate the neurobiological bases of sex differences in neural dynamics underlying cognitive decline.

## 3 RESULTS

### 3.1 Evolution of task scores with age

EEG recordings were collected during three‐choice vigilance task^26^, ^27^ execution from 119 SCD patients, along with 19 healthy subjects in the CTR group. Groups were homogenous in terms of demographics like age, sex, and education levels (see Table 1).

**TABLE 1:** Demographics, cognitive scores, and Task scores across groups (CTR and SCD).

	Groups		Test			
	**CTR**	**SCD**	**Test**	***p*‐value**	**ES**	**SC**
Age	62.5 [56.8, 71.6]	65.4 [47.85, 82.05]	U = 833	0.24	0.18	1.00
Sex	8F/11M	85F/44M	*χ* ^2^ = 3.05	0.080	0.09	0.83
Education	14.8 [10.4, 19.6]	13.4 [7.25, 18.0]	U = 1114	0.22	0.10	1.02
MMSE	29 [27, 30]	28 [23, 30]	**U = 1286**	**0.011**	**0.27**	**2.68**
Cognitive reserve	‐	113 [103, 121]	‐	‐	‐	‐
Reaction time	0.44 [0.33, 0.61]	0.46 [0.34, 0.64]	U = 878	0.38	0.13	0.81
Accuracy	0.95 [0.89, 0.99]	0.94 [0.82, 0.99]	U = 1112	0.46	0.10	0.74
F‐measure	0.91 [0.84, 0.96]	0.89 [0.77, 0.96]	U = 1183.5	0.26	0.17	0.97
Age at onset	‐	57.0 [36.2, 74.0]	‐	‐	‐	‐

*Note*: Bold values in the Test column represent statistically significant differences. Numerical quantities were compared with the Mann–Whitney U‐test, while categorical quantities were compared with the *χ* ^2^ test. Standardized effect sizes are reported (ES in the table). Group size corrections (SC in the table) are computed with Bayesian factor to account for uneven group size. Quantities between brackets represent confidence intervals with 95% confidence level. Colors represent diagnostic groups (blue for CTR and red for SCD).

Abbreviations: CTR, control; ES, effect size; MMSE, Mini‐Mental State Examination; SC, size correlation; SCD, subjective cognitive decline.

The CTR group scored higher in the Mini‐Mental State Examination (MMSE) compared to the SCD group (U = 1286, *p* = 0.011, EF = 0.27, BF = 2.68), while no statistically significant differences were found between the CTR and SCD groups in terms of Task Accuracy, Task Reaction Time, and F‐Measure (Table 1).

An examination of the relationship between Task Reaction Time and Task Accuracy revealed a significant positive correlation in the CTR group (*r* = 0.52, *p* = 0.033, BF = 1.23, Figure S1A). Interestingly, this correlation was absent in the SCD group (*r* = –0.03, *p* = 0.76, Figure S1B), indicating a divergence in how CTR subjects and SCD patients behaved during the task, corroborated by Fisher *Z*‐test (*p* = 0.020, BF = 1.22). Similarly, no correlations between Task Accuracy and Task Reaction Time were found in sex SCD subgroups, with both SCD Females and SCD Males presenting statistically significant differences (albeit with weak effect size) with respect to the correlation found in CTR subjects (Fisher *Z*‐test, respectively *p* = 0.049, BF = 0.72 and *p* = 0.033, BF = 1.18).

We then analyzed how cognitive scores and Task scores correlated with participants’ age across groups. In both CTR and SCD participants, MMSE values presented no correlation with age (respectively *r* = 0.13, *p* = 0.62; *r* = –0.03, *p* = 0.91). Cognitive Reserve (measured only in SCD participants) significantly increased with age (*r* = 0.26, *p* = 0.013, BF = 1.79). Also, education presented significant differences between CTR and SCD in its relationship with patient's age (Fisher *Z*‐test, *p* = 0.0005, BF = 3.15). While in CTR subjects the two quantities were positively correlated (*r* = 0.57, *p* = 0.018, BF = 1.52), in SCD patients the correlation was negative (*r* = –0.34, *p* = 0.0001, BF = 3.06).

Task Accuracy significantly decreased with age in SCD patients (*r* = –0.28, *p* = 0.002, BF = 2.09), while no significant age‐related decline was found in the CTR group (*r* = –0.08, *p* = 0.76, Figure S2A). Similarly, Task Reaction Time increased with age in SCD patients (*r* = 0.33, *p* = 0.0001, BF = 2.95, Figure S2B), but not in CTR subjects (*r* = 0.26, *p* = 0.31). These two effects combined determined the overall task proficiency ‐measured with the F‐measure‐ to significantly decrease with age in SCD patients (*r* = –0.41, *p* < 0.00001, BF = 4.72) but not in CTR subjects (*r* = –0.36, *p* = 0.16). However, these differences were not significant after Fisher *Z*‐test.

### 3.2 Task scores significantly decline with age only in female SCD patients

To investigate sex‐specific patterns in cognitive decline, we analyzed SCD patients stratified according to their biological sex. Since CTR Females and CTR Males presented no differences in demographics, cognitive, and task scores (Table S2), we considered CTR participants a unique group. SCD sex subgroups instead presented relevant differences, which can be consulted in Table S3.

**FIGURE 2:**
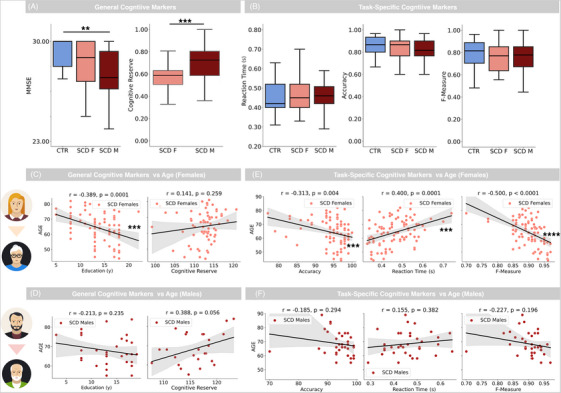
Cognitive and task scores and their correlation with age across sex subgroups. (A) Left: Boxplot showing MMSE values in CTR subjects (light blue), SCD Females (salmon), and SCD Males (dark red). Right: Boxplot showing Cognitive Reserve values in SCD Females and SCD Males. Cognitive Reserve values are normalized. Whiskers in the boxplot are set to 1.5 times interquartile range values; median value is represented by a black horizontal line. (B) Boxplot of task scores (left: Reaction Time; middle: Accuracy; right: F‐Measure) in CTR subjects, SCD Females, and SCD Males. Notation and color code are the same as in (A). (C) Correlation of participants’ age with years of education (left) and Cognitive Reserve (right) for SCD Females. Shaded area represents 2.5%–97.5% confidence interval. (D) Correlation of participants’ age with years of education (left) and Cognitive Reserve (right) for SCD Males. Notation is the same as in (C). (E) Correlation of participants’ age with task scores (left: Accuracy; middle: Reaction Times; right: F‐Measure) for SCD Females. Notation is the same as in (C). (F) Correlation of participants’ age with task scores (left: Accuracy; middle: Reaction Times; right: F‐Measure) for SCD Males. Notation is the same as in (C). Significance notation * stands for *p* < 0.05, ** stands for *p* < 0.01, *** stands for *p* < 0.005, **** stands for *p* < 0.00001. CTR, control; MMSE, Mini‐Mental State Examination; SCD, subjective cognitive decline.

Analyzing general cognitive scores (Figure 2A), SCD Females presented MMSE values that did not differ significantly from CTR values (U = 711.5, *p* = 0.062, EF = 0.12), while SCD Males had significantly lower values, despite presenting low effect size (U = 418.5, *p* = 0.004, EF = 0.45, BF = 3.64). Differences between SCD Females and SCD Males were not significant (U = 1420.5, *p* = 0.30, EF = 0.02). Conversely, significant differences between SCD Females and SCD Males were found in Cognitive Reserve, with the latter showing significantly higher values (U = 412.5, *p* = 0.0002, EF = 0.78, BF = 5.88). Task scores presented no statistically significant differences across CTR, SCD Females, and SCD Males (Figure 2B, values are reported in Table S3).

We then analyzed how general cognitive scores correlated with age, finding that MMSE presented no significant correlation in both SCD Females (*r* = –0.03, *p* = 0.79) and SCD Males (*r* = 0.19, *p* = 0.29). Conversely, education decreased with age in both sex subgroups, albeit the correlation was statistically significant for SCD Females (*r* = –0.39, *p* = 0.0001, BF = 3.01, Figure 2C) but not for SCD Males (*r* = –0.21, *p* = 0.23, Figure 2D). Moreover, Cognitive Reserve increased with age in both subgroups, although non‐significantly (Females: *r* = 0.14, *p* = 0.26, Figure 2C; Males: *r* = 0.39, *p* = 0.056, Figure 2D).

We then analyzed how Task scores evolved with age in the SCD sex subgroups (remembering that these quantities presented no correlation with age in the CTR group). Task Accuracy was found to decrease significantly with age in SCD Females (*r* = –0.31, *p* = 0.004, BF = 1.84, Figure 2E), while no significant trend was observed in SCD Males (*r* = –0.18, *p* = 0.29, Figure 2F). Similarly, Task Reaction Time increased with age in SCD Females (*r* = 0.40, *p* = 0.0001, BF = 3.18, Figure 2E) but remained stable in SCD Males (*r* = 0.15, *p* = 0.38, Figure 2F). As a result, the F‐measure, which reflects overall task proficiency, showed a pronounced decline with age in SCD Females (*r* = –0.50, *p* < 0.00001, BF = 5.25, Figure 2E), while no significant decline was observed in SCD Males (*r* = –0.23, *p* = 0.20, Figure 2F). Differences regarding the correlation of Task scores with age were not significant after Fisher' *Z*‐test.

### 3.3 SCD patients present sex‐specific differences in ERP features

We then analyzed neural markers derived from ERPs extracted during task execution (see Section 2), investigating the maximum amplitude, the integral and the latency of ERP components. Neural marker analyses showed strong group differences in some of the ERP components, with the CTR group exhibiting significantly larger N1 integral and maximum amplitude values and higher P900 maximum amplitude values compared to the SCD group. Group‐wise differences in ERP features are reported in Table S4.

To assess the interactions of neural markers with cognitive and task scores, we ran a correlation analysis between these quantities, for both CTR and SCD groups. No relationship between ERP features and Task scores was found in either the CTR nor the SCD group. Similarly, no relevant correlation between ERP features and age was found between CTR and SCD groups, except for the N1 component, whose integral values significantly correlated with participants’ age in the CTR group (*r* = 0.56, *p* = 0.021, BF = 1.47, Figure S3A) but not in the SCD group (*r* = 0.05, *p* = 0.57). This difference was confirmed by Fisher *Z*‐test (*p* = 0.044). In the SCD group, instead, the N1 latency increased with age (*r* = 0.33, *p* < 0.00001, BF = 2.10, Figure S3B), while this trend was not observed in the CTR group (*r* = 0.13, *p* = 0.62), although the difference between groups was not statistically significant.

**FIGURE 3:**
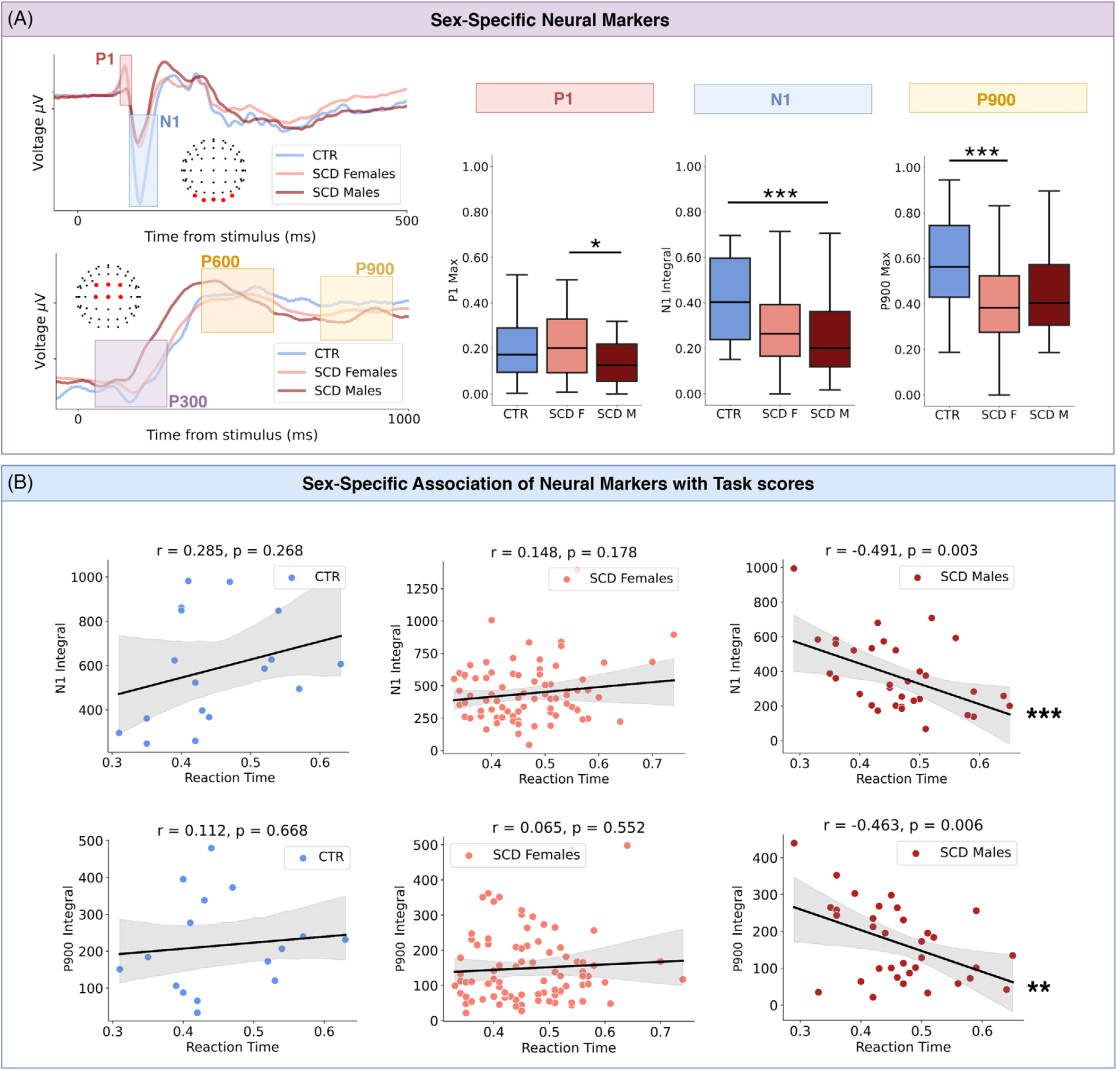
Sex‐specific differences in ERP features. (A) Top left: Average waveforms from occipital channels for CTR (light blue) SCD Females (salmon) and SCD Males (dark red). P1 and N1 component are highlighted. Bottom left: Average waveforms from occipital channels for CTR SCD Females and SCD Males. P300, P600, and P900 components are highlighted. Right: Boxplot showing the P1 maximum amplitude values, the N1 integral values and the P900 maximum amplitude values in the three groups. Whiskers in the boxplot are set to 1.5 times interquartile range values; median value is represented by a black horizontal line. (B) Top: Correlation between the N1 component integral and Task Reaction Time across groups: CTR on the left, SCD Females in the middle and SCD Males on the right. Bottom: Correlation between the P900 component integral and Task Reaction Time across groups: CTR on the left, SCD Females in the middle and SCD Males on the right Shaded area represents 2.5%–97.5% confidence interval. Significance notation: Significance notation * stands for *p* < 0.05, ** stands for *p* < 0.01, *** stands for *p* < 0.005. CTR, control; ERP, event‐related potential; SCD, subjective cognitive decline.

We then analyzed sex‐specific differences in ERP features (Figure 3A and Table 2). In the encoding phase, the P1 component presented significantly higher values in SCD Females than in SCD Males (U = 1881, *p* = 0.010, ES = 0.30, BF = 2.81). For the N1 component, both SCD Males and SCD Females showed reduced N1 maximum amplitudes and integrals compared to CTR. While the differences with respect to the CTR group in terms of N1 maximum amplitudes were significant for both SCD Females and SCD Males (respectively: U = 433, *p* = 0.010, EF = 0.46, BF = 2.93; U = 130, *p* = 0.002, EF = 0.55, BF = 4.80), differences in terms of the N1 integral were significant only between CTR and SCD Males (U = 440, *p* = 0.003, EF = 0.52, BF = 4.22), while differences between CTR and SCD Females were statistically significant but did not survive Bonferroni correction (U = 973, *p* = 0.025, EF = 0.20, BF = 2.26).

**TABLE 2:** ERP features across groups (CTR and SCD) groups (CTR, SCD Females, and SCD Males).

		**CTR**	**SCD F**	**SCD M**	**Test**	***p*‐value**
P1	P1 Max [mV]	2.56 ± 0.49	2.73 ± 0.21	1.80 ± 0.23	**6.47**	**0.039**
	P1 Integral	186 ± 25	170 ± 10	132 ± 14	**6.21**	**0.045**
	P1 Latency [s]	68 ± 4	73 ± 2	73 ± 4	1.50	0.47
N1	N1 Max [mV]	7.91 ± 1.03	5.09 ± 0.40	4.22 ± 0.52	**10.20**	**0.006**
	N1 Integral	583 ± 60	439 ± 24	371 ± 35	**10.36**	**0.005**
	N1 Latency [s]	127 ± 3	125 ± 3	118 ± 4	1.65	0.44
P300	P300 Max [mV]	2.20 ± 0.30	2.20 ± 0.16	1.73 ± 0.22	4.59	0.11
	P300 Integral	347 ± 33	304 ± 19	266 ± 30	5.97	0.051
	P300 Latency [s]	415 ± 8	409 ± 4	413 ± 6	0.54	0.76
P600	P600 Max [mV]	2.03 ± 0.19	1.63 ± 0.11	1.57 ± 0.19	3.67	0.16
	P600 Integral	332 ± 34	272 ± 16	253 ± 29	4.43	0.11
	P600 Latency [s]	596 ± 16	600 ± 7	621 ± 12	2.09	0.35
P900	P900 Max [mV]	1.52 ± 0.19	0.93 ± 0.07	1.10 ± 0.11	**9.14**	**0.010**
	P900 Integral	214 ± 30	149 ± 11	168 ± 18	4.92	0.085
	P900 Latency [s]	872 ± 11	886 ± 5	878 ± 9	1.30	0.52

*Note*: Comparisons between the three groups were made by Kruskal–Wallis H‐test. Bold values in the Test column represent statistically significant differences. Colors in the first column represent different ERP components. Colors in other columns represent diagnostic groups (blue for CTR, salmon for SCD Females and dark red for SCD Males).

Abbreviations: CTR, control; ERP, event‐related potential; SCD, subjective cognitive decline.

The P300 and P600 components presented no significant group differences. Regarding the P900 component, SCD Females showed significantly lower maximum amplitudes compared to CTR (U = 1046, *p* = 0.004, EF = 0.33, BF = 3.66), while the difference between CTR and SCD Males did not reach significance after Bonferroni correction (U = 388, *p* = 0.049, EF = 0.34, BF = 1.84).

We then repeated the analysis correlating ERP features with Task scores for the SCD sex subgroups (Figure 3B). We found that Task Reaction Time, which presented no correlation with the N1 component integral values in CTR (*r* = 0.28, *p* = 0.27) and SCD Females (*r* = 0.15, *p* = 0.18), instead presented a significant anti‐correlation in SCD Males (*r* = –0.49, *p* = 0.003, BF = 1.96). Correlation of the N1 component integral values with Task Reaction Time was significantly different between CTR and SCD Males (Fisher *Z*‐test, *p* = 0.010, BF = 1.43), and between SCD Females and Males (Fisher *Z*‐test, *p* = 0.001, BF = 2.31). Similarly, P900 integral values presented no correlation with Task Reaction Time in CTR (*r* = 0.11, *p* = 0.67) and SCD Females (*r* = 0.06, *p* = 0.55), but they presented a significant anti‐correlation in SCD Males (*r* = –0.46, *p* = 0.006, BF = 1.70). While differences between CTR and SCD Males were not significant, the difference between SCD Females and SCD Males regarding the relationship of the P900 component integral values with Task Reaction Time values was significant (Fisher *Z*‐test, *p* = 0.007, BF = 1.52).

The P900 component integral values were the only quantity that presented a significant correlation with age in one of the SCD subgroups. In fact, while CTR presented no correlation (*r* = 0.31, *p* = 0.23, Figure S4A) SCD Females presented a significant correlation between the two quantities (*r* = 0.29, *p* = 0.007, BF = 1.60, Figure S4B), SCD Males displayed an opposite trend, with a non‐significant anti‐correlation (*r* = –0.22, *p* = 0.22, Figure S4C). Statistical test confirmed the significance of the difference between SCD Females and SCD Males regarding the relationship of the P900 integral values with age (Fisher *Z*‐test, *p* = 0.014, BF = 1.33).

### 3.4 ERP features improve SCD diagnostic performance and remove sex imbalances

**FIGURE 4:**
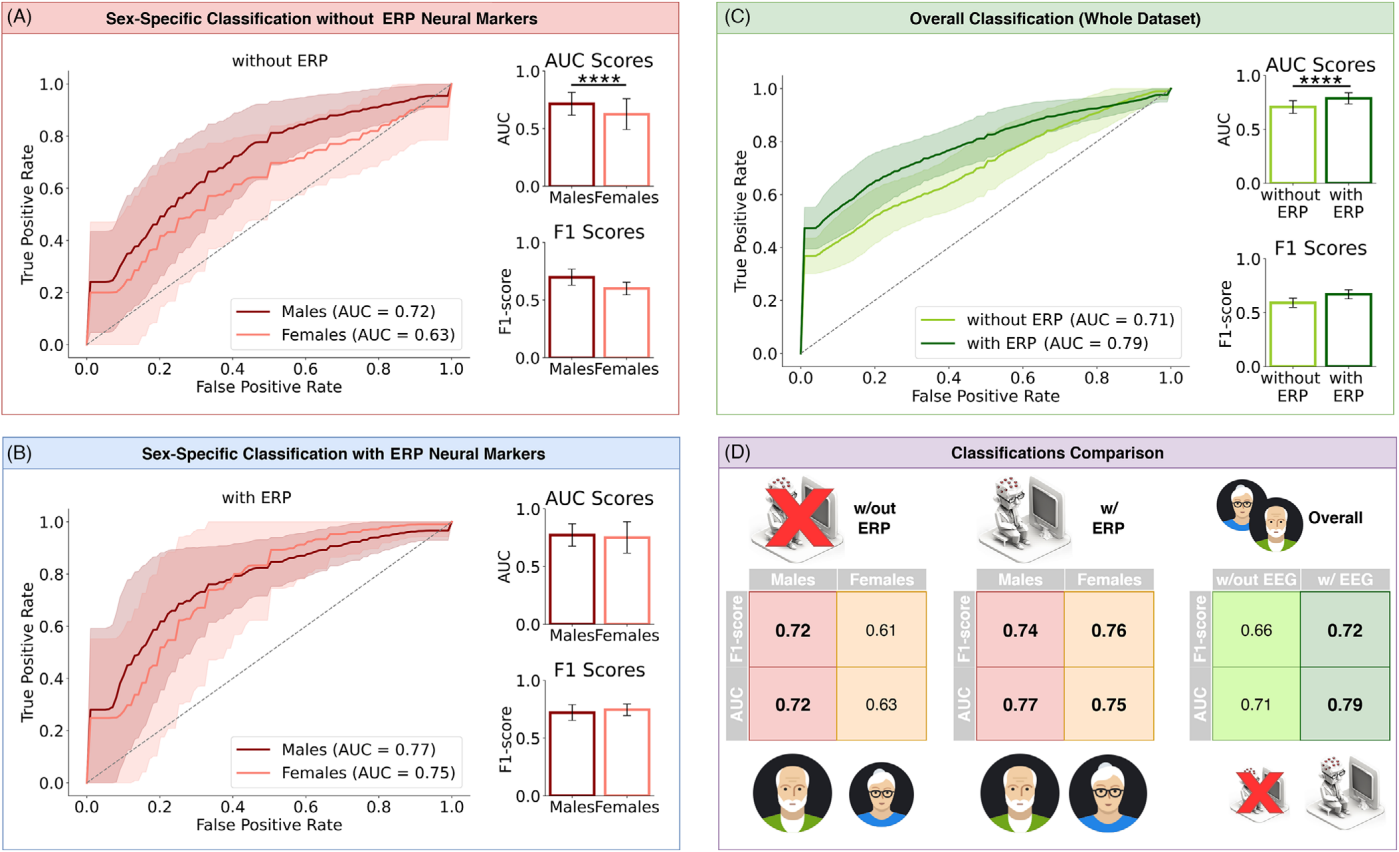
ERP features significantly improve classification performances, particularly in female participants. (A) ROC curves and classification scores (AUC and F1 score) for the sex‐specific classifications between CTR and SCD participants, made without ERP features. SCD Males and SCD Females are reported in dark red and salmon, respectively. (B) ROC curves and classification scores (AUC and F1 score) for the sex‐specific classifications between CTR and SCD participants, made including ERP features. Notation is the same as in (A). (C) ROC curves and classification scores (AUC and F1 score) for the overall (whole dataset) classifications between CTR and SCD participants, comparing results with and without ERP features. Results obtained using ERP features are reported in dark green, while results without ERP features are reported in yellow green. (D) Summary of classification scores in the previous panels, showcasing the added value of ERP features. Significance notation: **** stands for *p* < 0.00001. AUC, area under the curve; CTR, control; ERP, event‐related potential; ROC, receiver operating characteristics; SCD, subjective cognitive decline.

We investigated whether ERP features could enhance the machine‐learning diagnosis of SCD, that is, the classification between CTR and SCD participants. Classifications were performed using a Logistic Regression algorithm with nested cross‐validation (see Section 2). To assess the sex‐specificity of the ERP features, we first performed sex‐specific classifications: CTR Males versus SCD Males and CTR Females versus SCD Females. We first performed the classification using only non‐neural clinical features including age, Cognitive Reserve, MMSE scores, years of education, Task Reaction Time, Task Accuracy, and F‐measure. This classification presented substantially better performance for males than for females. Specifically, male classification reached an F1 score of 0.72 ± 0.07 and an AUC of 0.72 ± 0.10, while female classification showed significantly lower values, with an F1 score of 0.61 ± 0.06 and an AUC of 0.63 ± 0.13. SCD Males had a higher probability of being correctly classified with respect to SCD Females (respectively 70% vs. 49%). The difference in classification performance between sexes was highly significant (Mann–Whitney U‐test, *p* < 0.00001, Figure 4A, effect size *d* = 0.8), showing a strong sex‐related bias in models trained without using ERP features.

We then repeated the sex‐specific classification by including ERP features (see Section 2) as additional classifying features. Crucially, the inclusion of these neural markers improved classification performance in both sexes and removed the previous imbalance. The new models yielded an F1 score of 0.74 ± 0.07 and an AUC of 0.77 ± 0.10 for males, and an F1 score of 0.76 ± 0.05 and an AUC of 0.75 ± 0.14 for females. Notably, SCD Males and SCD Females presented a similar probability of being correctly classified (respectively 67% vs. 69% probability). Performance difference between sexes was no longer significant (Mann–Whitney U‐test, *p* = 0.31, Figure 4B).

Finally, we repeated the classification for the whole dataset, regardless of biological sex. We attempted the classification both without and with the ERP features. When considering the entire dataset irrespective of sex, we found that classifiers including ERP features significantly outperformed those based solely on non‐neural clinical features. The model with ERP features achieved an F1 score of 0.72 ± 0.04 and an AUC of 0.79 ± 0.06, compared to F1 score = 0.66 ± 0.02 and AUC = 0.71 ± 0.05 without EEG (Mann–Whitney U‐test, *p* < 0.00001, Figure 4C, with a large effect size *d* = 1.4). These results confirm the added value of ERP features for early diagnosis, particularly for the correct classification of SCD Females (Figure 4D).

To explicitly account for class imbalance, a balanced subsampling strategy was adopted for all classification tasks. Results are reported in Supporting Information (Figure S5).

## 4 DISCUSSION

In this study, we employed ERP features to uncover sex‐specific mechanisms associating neural activity during task execution with Task scores, providing a sex‐unbiased SCD diagnosis.

Task scores, such as Task Accuracy and Task Reaction Time, showed a clear degradation with age, but only in the SCD group. Older CTR subjects performed on par with younger CTRs, indicating that in the absence of underlying pathology, aging (in the considered interval) does not necessarily lead to cognitive decline.^31^ This suggests that cognitive performance is maintained in healthy aging, while the presence of SCD is associated with age‐related cognitive deterioration. The lack of significant differences in cognitive performance across age values in the CTR group underscores the importance of identifying pathological changes early,^32^ as normal aging does not inherently lead to cognitive deficits.

A particularly interesting finding was that the pronounced degradation of Task scores with age in the SCD group was significant only in female patients. This sex‐specific decline presented an interplay with education levels, indicating that older females, who were generally less educated, appeared more vulnerable to cognitive decline. This suggests that social and cultural factors, such as educational opportunities, may contribute significantly to the observed sex differences in cognitive vulnerability.^6^, ^33^, ^34^ This link could be crucial for achieving equality in cognitive decline recognition. In fact, females usually show higher levels of global cognitive scores (that we also observed in this study, Figure 2A), potentially leading to higher levels of misdiagnosis. The use of cognitive task data could provide more robust markers of cognitive decline in females.

Importantly, our approach revealed sex‐specific neural markers of cognitive decline by analyzing ERP features recorded during task execution. We identified distinct components associated with SCD in females and males: the P900 component, which significantly discriminated SCD Females from control participants, and the N1 component, which differentiated SCD Males from controls. Moreover, we observed that the P1 component distinguished SCD Females from SCD Males, despite showing no difference when comparing all SCD participants to controls. This finding emphasizes that sex stratification is crucial for uncovering pathological alterations in neural activity that remain hidden in mixed cohorts.

Our study highlights sex‐specific alterations in the P1 and N1 ERP components associated with early cognitive decline, potentially indicating a broad involvement of brain regions governing visuo‐attentive processes.^35^ Several studies have hypothesized that P1 and N1 originate from neural circuits related to higher‐order cognitive processing, extending beyond the primary visual cortex. Specifically, P1 has been linked to extra‐striate visual encoding areas such as V2, V3, and V4,^36^ while N1 is potentially generated by regions along the occipital–parietal–temporal–frontal axis.^37^ Alterations in these components may also reflect abnormalities in stimulus‐driven facilitation within a neural pathway, leading to enhanced activation of the same pathway (benefit), as well as in the widespread inhibition exerted on neighboring regions (cost). Particularly, occipital P1 and N1 potentials are reported to encode (respectively) the cost and benefit of visual recognition.^38^, ^39^, ^40^

Our results also suggest alterations in the P900 component. P900 is a relatively new ERP component, first characterized by Rosenfeld et al.,^41^ and has been proposed to reflect countermeasures adopted by the subject while performing the task.^42^ Selective modulation of the P900 component during a visuo‐attentive task has been previously reported by our group,^27^ while alterations have also been observed during sleep and auditory tasks.^43^, ^44^

One of the key results of our analyses is that the inclusion of ERP features allowed our machine‐learning classifier to achieve an equitable SCD diagnosis, eliminating the sex‐bias inherent in clinical data alone (Figure 4). Despite growing interest in the intersection of machine learning and sex differences in AD, existing studies rarely explore sex‐specific mechanisms underlying the preclinical stages of cognitive decline, such as SCD. Moreover, to our knowledge, the use of ERP features in a sex‐specific classification framework has not been previously attempted.

Few machine learning studies have explicitly investigated sex‐specific differences in cognitive decline and dementia. Tang et al.^45^ identified sex‐specific patterns leading to AD progression leveraging large‐scale electronic records. Cieri et al.^46^ highlighted a sex‐specific association between memory performance and cortical thickness. Similarly, Sarica et al.^47^ confirmed that females have a higher risk of progressing to dementia compared to males using an array of biomarkers including glucose metabolism, cerebrospinal fluid, and magnetic resonance imaging (MRI) data. Among sex‐specific mechanisms, they found that hippocampal volume predicted future AD conversion in males, while verbal memory and executive function were the strongest predictors in females. Notably, Klingenberg et al.^48^ observed better classification performance for females than for males when using MRI data. We observed an opposite male‐biased diagnosis when using only clinical data. However, our approach based on the inclusion of ERP features led to a sex‐unbiased SCD diagnosis. D'Amore et al.^49^ employed an explainable machine learning approach to classify patients with clinical cognitive decline in a sex‐specific manner, with classification performance differing significantly between sexes.

It is worth noting that the study presents several limitations. First, the sample sizes used for task‐EEG analysis are uneven. Notably, the numerosity of the CTR group is smaller than the SCD group. This has been tackled by computing group size corrections with metrics that explicitly account for the numerosity of different groups, and by conducting classifications with balanced resampling. However, future studies should explore larger and more balanced cohorts to ensure equal representation of different clinical profiles. Furthermore, the study primarily focuses on neural markers collected during a visuo‐attentive task, which offer a significant, but limited window into the vast array of neural functions affected by cognitive decline. Furthermore, most participants come from the same ethnic background, potentially introducing bias in our results.

In conclusion, the integration of ERP features in the assessment of cognitive decline revealed important sex‐specific patterns of cognitive decline, leading to an equitable identification of the condition. The inclusion of ERP features in the machine learning classifier provided a sex‐unbiased SCD diagnosis, which was impossible to obtain using only clinical data and task scores.

## CONFLICT OF INTEREST STATEMENT

The authors have no conflicts of interest to disclose. Author disclosures are available in the supporting information.

## CONSENT STATEMENT

We confirm that all human subjects provided informed consent and that the study followed ethical standards (see also Supporting Information Methods).

## Supporting information



Supporting Information

Supporting Information
