# Supplementary material for: EEG recordings during visuo‐attentive task reduce sex bias in Alzheimer's disease diagnosis
Source: Alzheimers Dement (N Y). 2026 Apr 6;12(2):e70245. doi: 10.1002/trc2.70245 (PMC13053929; doi:10.1002/trc2.70245)
Supplement: Supplementary file 1 — Supporting Information [file TRC2-12-e70245-s002.docx]

# Supplementary Materials

# Supplementary Methods

**Participants recruitment**

Participants were recruited as part of the PREVIEW project ^1^, a longitudinal study on SCD condition as a prodromal form of dementia. A total of 119 self-referring patients with cognitive complaints classified as SCD were included in this study. We included a control group of 19 healthy subjects that volunteered for the study. The SCD group presented a 2 to 1 females to male ratio, following the expected sex ratio of the condition ^2–4^, particularly evident for early stages of cognitive decline ^5^. Classification into cognitive categories was based on extensive genetic and behavioral tests, following the NIA-AA criteria for subjective cognitive decline and mild cognitive impairment diagnosis ^6^. Inclusion criteria and ethical standards are specified in Supplementary Materials and can be consulted in the clinical trial site of the PREVIEW project.

**Cognitive Task and Task scores**

Participants underwent a cognitive task battery with concurrent EEG recording, executing a three-choice Vigilance Task (3CVT) test, tailored to evaluate sustained attention and cognitive performance by recognizing a target shape among two confounders. The task architecture is extensively discussed in Supplementary Methods. Task performance was assessed based on Task Accuracy (fraction of correct responses) and Task Reaction Time (average time between stimulus presentation and response). An additional metric called F-Measure ^7^, combining the average velocity and correctness of responses, was also included.

**Cognitive performance metrics**

An extensive neuropsychological evaluation preceded the classification of participants into cognitive categories. Global cognitive status was assessed using the Mini-Mental State Examination ^8^ (MMSE). Cognitive Reserve was estimated using the Test di Intelligenza Breve ^9^ (TIB), an Italian version of the National Adult Reading Test (NART) ^9,10^. TIB values were extracted in 91 SCD patients only (66F / 25M), so analysis on TIB values are restricted to that subset. Extensive information on the neurophysiological test battery and on diagnostic procedure can be found in the clinical trial website of the PREVIEW project ^11^.

**Inclusion criteria and ethical standards**

Inclusion criteria consisted of: (i) meeting diagnostic criteria for SCD condition; (ii) Mini Mental State Examination (MMSE) score >24, adjusted for age and years of education; (iii) normal scores on the Activities of Daily Living (ADL) and the Instrumental Activities of Daily Living (IADL) scales. Exclusion criteria included: (i) history of severe head traumas; (ii) presence of neurological and/or systemic diseases, psychosis, or major depression; (iii) history of substance abuse (including alcohol); (iv) past or current use of medications known to interact with EEG oscillations (e.g., benzodiazepines, antiepileptic drugs). Additionally, subjects meeting criteria for MCI or overt AD diagnosis according to the National Institute on Aging-Alzheimer’s Association (NIA-AA) were excluded.

Participant recruitment and EEG recordings adhered to the guidelines set forth by the Declaration of Helsinki and the standards outlined by the Committee on Human Experimentation of the Careggi University Hospital in Florence, Italy. The study received approval from the local Institutional Review Board (reference 15691oss). Relevant demographic information about participants is provided in Supplementary Table 2 and Supplementary Table 3. EEG acquisitions started on October 2022, while the analyses here discussed were made on patients included in the dataset as of January 2025.

**Cognitive Task Architecture**

The 3CVT task required participants to discriminate among three geometric shapes: a primary upward triangular target (referred to as TARGET) and two secondary diamond and inverted triangular targets (referred to as NON-TARGET). The task duration was set at 20 minutes. During the task, each shape appeared individually at 200 ms intervals, followed by an 800 ms window for the response. Trials were interspersed with breaks of random duration between presentations. Participants responded using a keypad, indicating whether the stimulus presented was a TARGET (left button) or a NON-TARGET (right button). TARGET stimuli occurred with a 70% probability, while the two NON-TARGET stimuli were presented with a 30% probability. A brief training session preceded the test to mitigate errors due to unfamiliarity with the task. The test concluded with the presentation of a black screen. Total number of trials per participant was 421±78.

**Experimental EEG pre-processing and ERP computation**

EEG data were preprocessed in the MATLAB plugin EEGLAB. After removing non-EEG channels (ECG, EOG, A, B, MK), the data consisted of 61 scalp EEG channels, which were assigned standard 10–20 electrode locations and band-pass filtered between 1 and 45 Hz. Continuous data were cleaned using the clean_rawdata pipeline (FlatlineCriterion = 5 s; ChannelCriterion = 0.8; LineNoiseCriterion = 4; Highpass = off; BurstCriterion = 20; WindowCriterion = 0.25; BurstRejection = off; Distance = Euclidian; WindowCriterionTolerances = [−Inf, 7]). Channels identified as bad by clean_rawdata were interpolated using spherical interpolation, and the data were subsequently re-referenced to the common average. Independent component analysis (runica, extended infomax = 1) was performed on the cleaned data, and components were automatically classified using ICLabel. All components whose highest-probability class resulted to be different from “Brain” (i.e., Muscle, Eye, Heart, Line Noise, or Channel Noise) were removed prior to ERP computation. We additionally computed, for each group condition, the mean and standard deviation of the percentage of rejected ICA components, the number of bad channels and the number of retained trials. These quality-control metrics are reported in Supplementary Table 1.

Each trial of the 3-CVT task experiment lasted 1000ms, divided into two parts: stimulus presentation in the first 200 ms, and participant response in the subsequent 800ms.

ERPs were epoched in the [-100, 1000] ms window, aligning the 0 ms instant with stimulus presentation. We analyzed the timeframe encoding processing, which includes the P1 and N1 neural markers (respectively, the first positive and negative deflection after stimulus onset with the P1 usually centered in the [50, 100] ms window and the N1 usually centered in the [100, 150] ms window. Both the P1 and the N1 are recorded from occipital channels (PO7, PO8, O1, O2, Oz). P1 and N1 ERP amplitudes were determined by computing maximum N1 depths in the encoding timeframe. To investigate decision processing, we also analyzed the [300 ms, 1000 ms] window in central channels (FC1, FCz, FC2, C1, Cz, C2), associated with the P300 (usual window: [300 ms, 500 ms]), P600 (usual window: [500 ms, 750 ms]) and P900 (usual window: [800 ms, 1000 ms]) components. Neural markers were extracted from ERP components by computing the integral, maximum amplitude and latency (defined as the time of maximum amplitude) in their respective windows, and averaged across trials.

**Machine Learning Pipeline**

To evaluate the diagnostic potential of task-related EEG neural markers, we implemented a machine learning classification pipeline using a Logistic Regression approach. Given class imbalance and the need for robust generalization estimates, we adopted a nested cross-validation strategy, with an inner k-fold (k=3) cross-validation used for hyperparameter tuning and an outer Leave-One-Out (LOO) cross-validation used for performance evaluation. To ensure balanced learning, we enabled class-weight balancing in the classifier. This setup ensured that model tuning was performed independently of the test sample, minimizing overfitting.

Classification performance was assessed using several metrics. For each LOO fold, binary predictions (0 or 1) were generated by applying a 0.5 probability threshold. We computed F1-score per fold, then averaged them across folds to obtain the overall mean and standard deviation. Given the limitations of AUC calculation on single samples, we instead accumulated predictions and ground-truth labels across all LOO folds, and then computed the overall Area Under the ROC Curve (AUC).

To visualize the classification performance, we constructed the Receiver Operating Characteristic (ROC) curve from the aggregated predictions. Confidence intervals for the ROC curve were estimated using a percentile bootstrap method with 1000 resamplings. For each bootstrap iteration, we sampled prediction-label pairs with replacement and recalculated the ROC curve. Confidence bands were then derived from the 2.5th and 97.5th percentiles of the bootstrap distribution at each false positive rate.

Two separate classification scenarios were tested: A classification involving only clinical and cognitive scores and a classification also involving ERP features. In the clinical-only classification, candidate features included: age, MMSE, education, Task Reaction Time, Task Accuracy, and F-Measure. In the clinical plus task-EEG classification, we added neural markers extracted from ERPs (Table 2). Both integral values and maximum amplitude values were used in the classification, while latencies were omitted since they presented no significant differences across any of the included ERP components. All features were z-scored prior to classification inside the nested-cross validation loop. To account for class imbalance between groups, we run machine learning classifications with balanced resamplings, checking consistency with the results obtained with the standard classifications. Results are reported in the Main Text, while the methodology is described in the “*Correction for group imbalance: Classifications*” paragraph of Supplementary Materials.

**Correction for group imbalance: Statistical tests**

Standardized effect sizes were computed for all binary comparisons to account for group imbalance, using the formulas:

$$ES=1- \frac{2U}{n_{1}n_{2}}$$

For the Mann-Whitney, with $U$ being the statistic value and $n_{1}$ and $n_{2}$the group sizes.

For $\chi^{2}$ tests, the standardized effect size formula was:

$$ES= \sqrt{\frac{\chi^{2}}{n(k-1)}}$$

Where $n$ is the number of categories and $k$ the numerosity of the smallest group. To further account for differences in sample size between groups in statistical comparisons, we adopted a post-hoc Bayesian evidence-based framework to all significant binary comparisons. In addition to conventional non-parametric tests (Mann–Whitney U test for group comparisons and correlation analyses), evidential strength was quantified using Bayes factors (BF), which provide a direct measure of how strongly the observed data support the alternative hypothesis relative to the null hypothesis. For correlation analyses and comparisons between correlation coefficients, Bayes factors were derived from the corresponding Fisher Z-transformed statistics.

Unlike p-values, Bayes factors explicitly incorporate sample size through the likelihood function, thereby naturally accounting for differences in group numerosity. Larger sample sizes contribute greater evidential weight only insofar as they provide additional information, preventing artificial inflation of significance due to unbalanced group sizes. Log-transformed Bayes factors were interpreted according to standard conventions, with increasing values indicating progressively stronger evidence for the alternative hypothesis. To facilitate comparability across analyses, evidential strength was evaluated on the log scale rather than the raw BF scale, which can vary across orders of magnitude even for similar effect sizes. This approach allowed us to assess statistical evidence in a manner that is robust to group imbalance and directly comparable across heterogeneous analyses.

**Correction for group imbalance: Classifications**

To explicitly account for class imbalance in classification analyses, we implemented a two-level balancing strategy combining balanced subsampling during model training with post-hoc performance normalization. First, a balanced subsampling approach was applied to the training data: the majority class was randomly partitioned into multiple (n=10) non-overlapping subsets, each matched in size to the minority class. Each balanced dataset thus contained equal numbers of samples from both classes. Classification was then performed independently on each balanced subset using a LOO cross-validation scheme, with nested cross-validation for hyperparameter optimization. This procedure ensured that model training was not biased toward the majority class and that hyperparameter selection was performed exclusively on balanced data.

Performance metrics (F1 score and area under the ROC curve) and receiver operating characteristic curves were computed for each balanced subsample and subsequently averaged across repetitions, yielding estimates that are robust to sampling variability in the majority class. To further mitigate residual effects of class imbalance on performance estimates, a post-hoc balancing strategy was applied. Specifically, predictions obtained via LOO cross-validation after balanced subsampling were retained, and the majority class predictions were randomly partitioned into multiple (n=10) subsets matched in size to the minority class. For each balanced prediction set, classification metrics and ROC curves were recomputed and then averaged across repetitions.

This two-step procedure ensured that both model training and performance evaluation were minimally influenced by class imbalance, while preserving the full set of cross-validated predictions. By combining balanced training with post-hoc normalization of performance metrics, we obtained classification results that are stable, interpretable, and comparable across feature sets despite substantial differences in class size.

**Supplementary Results**

**Balanced resampling of classification tasks**

A balanced subsampling strategy was adopted to account for class imbalance between the CTR and SCD groups. All classification tasks were repeated to check for consistency with results reported in the main text. The majority class was randomly partitioned into multiple subsets matched in size to the minority class (see Supplementary Materials). For each balanced dataset, classification was performed using leave-one-out cross-validation with nested hyperparameter optimization. Performance metrics and ROC curves were subsequently averaged across repetitions. To further mitigate the effect of class imbalance on performance metrics, a post-hoc balancing strategy was applied. Predictions obtained via leave-one-out cross-validation (after balanced subsampling) were retained, and the majority class was randomly partitioned into multiple subgroups matched in size to the minority class. Classification metrics and ROC curves were computed for each balanced subset and subsequently averaged.

Results confirmed the findings of the previous analyses: while the classification based only on clinical data presented significant differences between females and males (females: F1 score=0.50±0.07, AUC=0.51±0.04; males: F1 score=0.71±0.04, AUC=0.69±0.05; p=0.0002) the classification based on ERP features presented no relevant differences (females: F1 score=0.74±0.03, AUC=0.77±0.05; males: F1 score=0.73±0.03, AUC=0.73±0.04; p=0.52). In classifications involving the whole cohort, the model which included ERP features presented significantly better performances compared to the model based on clinical data only (respectively: F1 score=0.71±0.02, AUC=0.74±0.03; F1 score=0.67±0.03, AUC=0.64±0.03; p=0.0002). Results are visualized in Supplementary Figure 5.

**Supplementary Tables and Figures**

|  | **Bad channels (#)** | | **Excluded ICA components (%)** | | | **Retained trials (#)** | |
| --- | --- | --- | --- | --- | --- | --- | --- |
|  | **mean** | **std** | **mean** | **std** | **mean** | | **std** |
| **CTR** | 8,68 | 4,89 | 55,70 | 9,95 | 428,84 | | 61,58 |
| **SCD** | 7,84 | 4,26 | 56,93 | 19,85 | 422,11 | | 82,38 |

**Supplementary Table 1:** **Data quality and preprocessing summary across experimental conditions**. Numbers of: (i) bad channels, (ii) excluded ICA components and (iii) retained trials are reported for the two groups. No statistical group difference was present across all measures.

|  | Groups | | MW Test | | | |
| --- | --- | --- | --- | --- | --- | --- |
|  | **CTR F** | **CTR M** | **Test** | **p-val** | **ES** | **SC** |
| **Age** | 62.1 [56.3, 70.6] | 62.8 [59.2, 71.8] | 42 | 0.90 | 0.15 | 0.59 |
| **Education** | 13.6 [10.2, 18.9] | 15.9 [13.0, 19.8] | 22.5 | 0.065 | 0.54 | 1.18 |
| **MMSE** | 29 [28, 30] | 29 [27, 30] | 45 | 0.95 | 0.09 | 0.31 |
| **Reaction Time** | 0.46 [0.39, 0.57] | 0.43 [0.32, 0.60] | 54.5 | 0.41 | 0.10 | 0.93 |
| **Accuracy** | 0.97 [0.93, 0.99] | 0.94 [0.89, 0.99] | 65.5 | 0.081 | 0.32 | 0.63 |
| **F-Measure** | 0.91 [0.87, 0.95] | 0.91 [0.84, 0.96] | 41.5 | 0.87 | 0.15 | 0.41 |

**Supplementary Table 2:** **Demographics, cognitive scores and task scores differences across CTR sex subgroups.** Standardized effect sizes are reported (ES in the table). Group size corrections (SC in the table) are computed with Bayesian Factor to account for uneven group size. Quantities between brackets represent confidence intervals with 95% confidence level.

#

|  | Groups | | | Tests | |
| --- | --- | --- | --- | --- | --- |
|  | **CTR** | **SCD F** | **SCD M** | **Test** | **p-val** |
| **Age** | 62.5 [56.8, 71.6] | 64.2 [45.3, 79.9] | 64.2 [55.0, 84.9] | H=5.27 | 0.073 |
| **Education** | 14.8 [10.4, 19.6] | 13.5 [7.8, 18.0] | 13.2 [7.0, 18.2] | H=1.52 | 0.47 |
| **MMSE** | 29 [27, 30] | 28 [23, 30] | 27 [23,30] | **H=7.87** | **0.019** |
| **Cognitive Reserve** | - | 112 [102, 118] | 116 [109, 122] | **U=412** | **0.0002** |
| **Reaction Time** | 0.44 [0.33, 0.61] | 0.47 [0.34, 0.64] | 0.46 [0.32, 0.64] | H=0.77 | 0.68 |
| **Accuracy** | 0.95 [0.89, 0.99] | 0.94 [0.82, 0.99] | 0.94 [0.84, 0.99] | H=1.29 | 0.52 |
| **F-Measure** | 0.91 [0.84, 0.96] | 0.90 [0.78, 0.96] | 0.90 [0.80, 0.96] | H=1.30 | 0.52 |
| **Age at onset** | - | 56.1 [36.8, 74.1] | 58.9 [44.3, 74.0] | U=1029.5 | 0.15 |

**Supplementary Table 3:** **Demographics, cognitive scores and task scores across groups (CTR, SCD Females and SCD Males).** Comparisons between three groups were made by Kruskal Wallis H-test, while binary comparison (only for Cognitive Reserve) were made with Mann Whitney U-test and comparison between categorical quantities were made with χ^2^ test. Bold values in the Test column represent statistically significant differences. Quantities between brackets represent confidence intervals with 95% confidence level.

#

#

|  | | Groups | | MW Test | | | |
| --- | --- | --- | --- | --- | --- | --- | --- |
|  |  | **CTR** | **SCD** | **Test** | **p-val** | **ES** | **SC** |
| **P1** | **P1 Max [mV]** | 2.56±0.49 | 2.47±0.17 | 1008 | 0.98 | 0.003 | 0.59 |
|  | **P1 Integral** | 186±25 | 158±9 | 1195 | 0.28 | 0.18 | 1.03 |
|  | **P1 Latency [s]** | 68±4 | 73±2 | 840 | 0.26 | 0.17 | 0.97 |
| **N1** | **N1 Max [mV]** | 7.91±1.03 | 4.84±0.32 | **563** | **0.003** | **0.44** | **3.75** |
|  | **N1 Integral** | 583±60 | 420±20 | **1413** | **0.008** | **0.40** | **3.00** |
|  | **N1 Latency [s]** | 127±3 | 123±3 | 1173.5 | 0.29 | 0.16 | 0.93 |
| **P300** | **P300 Max [mV]** | 2.20±0.30 | 2.06±0.13 | 1109 | 0.52 | 0.10 | 0.71 |
|  | **P300 Integral** | 347±33 | 293±16 | 1305 | 0.054 | 0.30 | 1.77 |
|  | **P300 Latency [s]** | 415±8 | 410±3 | 1075.5 | 0.67 | 0.06 | 0.64 |
| **P600** | **P600 Max [mV]** | 2.03±0.19 | 1.62±0.10 | 1285 | 0.072 | 0.27 | 1.60 |
|  | **P600 Integral** | 332±34 | 266±14 | 1301 | 0.057 | 0.29 | 1.74 |
|  | **P600 Latency [s]** | 596±16 | 606±6 | 935 | 0.62 | 0.08 | 0.66 |
| **P900** | **P900 Max [mV]** | 1.52±0.19 | 0.98±0.06 | **1434** | **0.006** | **0.42** | **3.32** |
|  | **P900 Integral** | 214±30 | 154±9 | 1316 | 0.045 | 0.30 | 1.87 |
|  | **P900 Latency [s]** | 872±11 | 884±4 | 883 | 0.40 | 0.13 | 0.80 |

**Supplementary Table 4: ERP features across groups (CTR and SCD).** Bold values in the Test column represent statistically significant differences. Standardized effect sizes are reported (ES in the table). Group size corrections (SC in the table) are computed with Bayesian Factor to account for uneven group size.


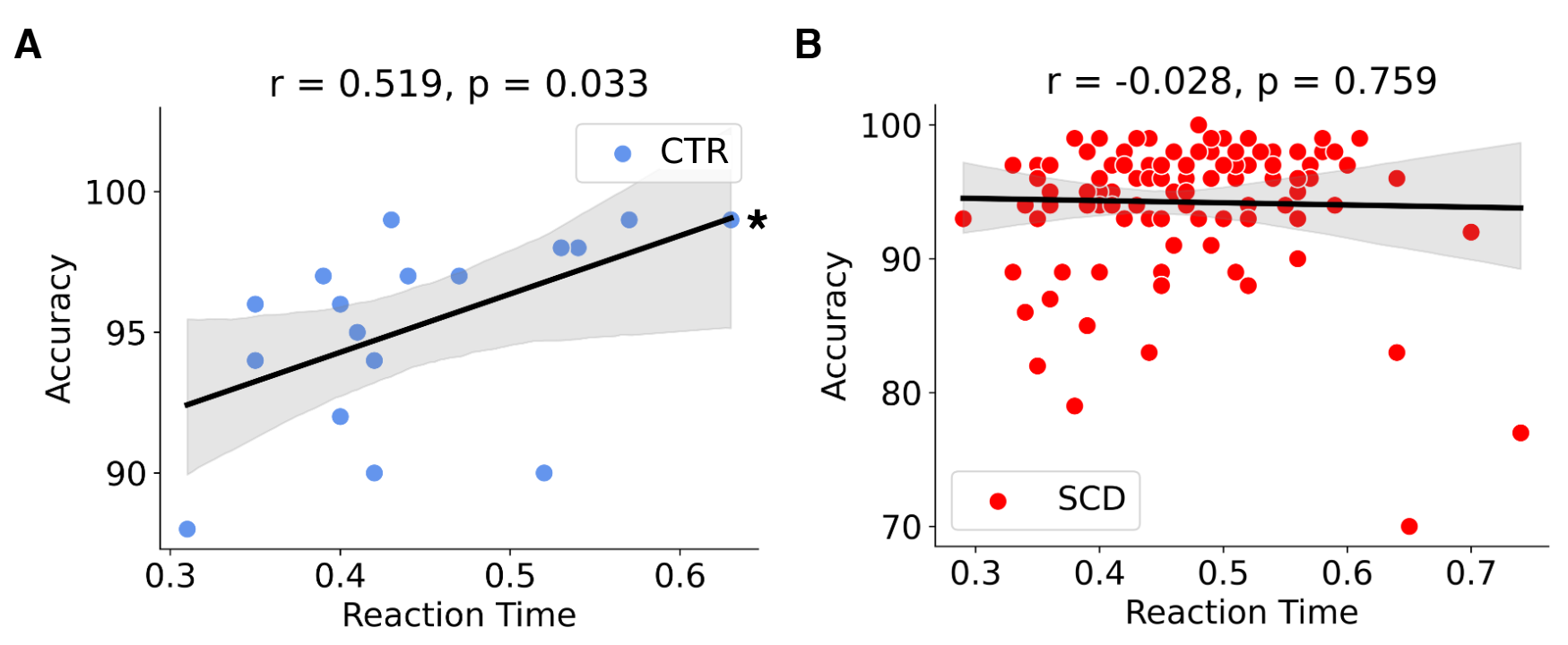


**Supplementary Figure 1: Task Accuracy correlates with Reaction Time in CTR participants only. (A):** Correlation between Task Accuracy and Task Reaction Time for CTR participants. Shaded area represents 2.5%-97.5% confidence interval. **(B):** Correlation between Task Accuracy and Task Reaction Time for SCD participants. Notation is the same as in (A). Significance notation: * stands for p<0.05.


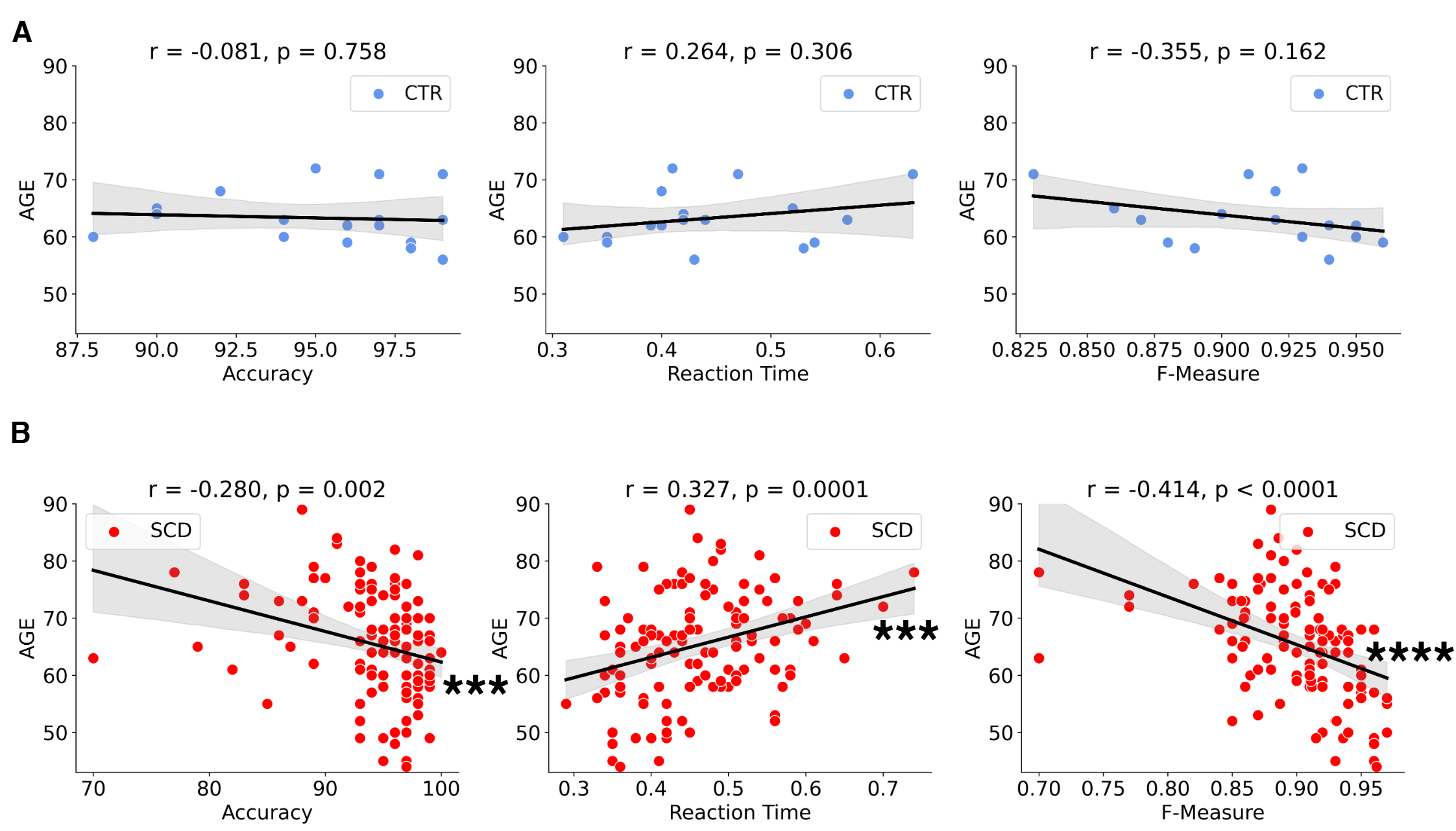


**Supplementary Figure 2: Task scores decrease with age in SCD patients. (A):** Correlation between Task scores and age in the CTR group. Shaded area represents 2.5%-97.5% confidence interval. **(B):** Correlation between Task scores and age in the SCD group. Notation is the same as in (A). Significance notation: *** stands for p<0.005, **** stands for p<0.00001.


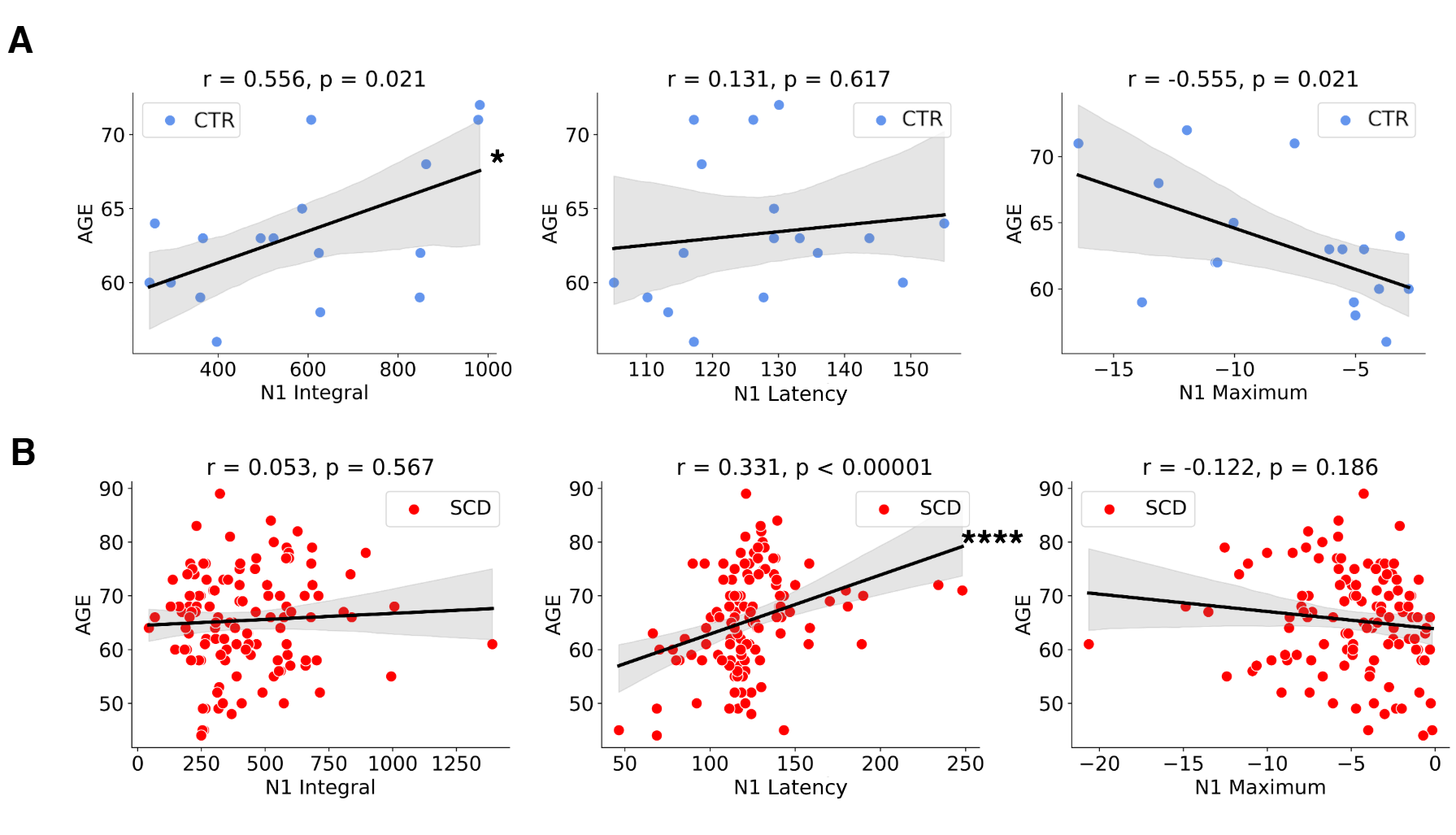


**Supplementary Figure 3: CTR and SCD participants present different evolution of the N1 component with age. (A):** Correlation between N1 integral values (left), latency values (middle) and maximum amplitude values (right) with age for the CTR group. Shaded area represents 2.5%-97.5% confidence interval. **(B):** Correlation between N1 integral values (left), latency values (middle) and maximum amplitude values (right) with age for the SCD group. Notation is the same as in (A). Significance notation: * stands for p<0.05, **** stands for p<0.00001.


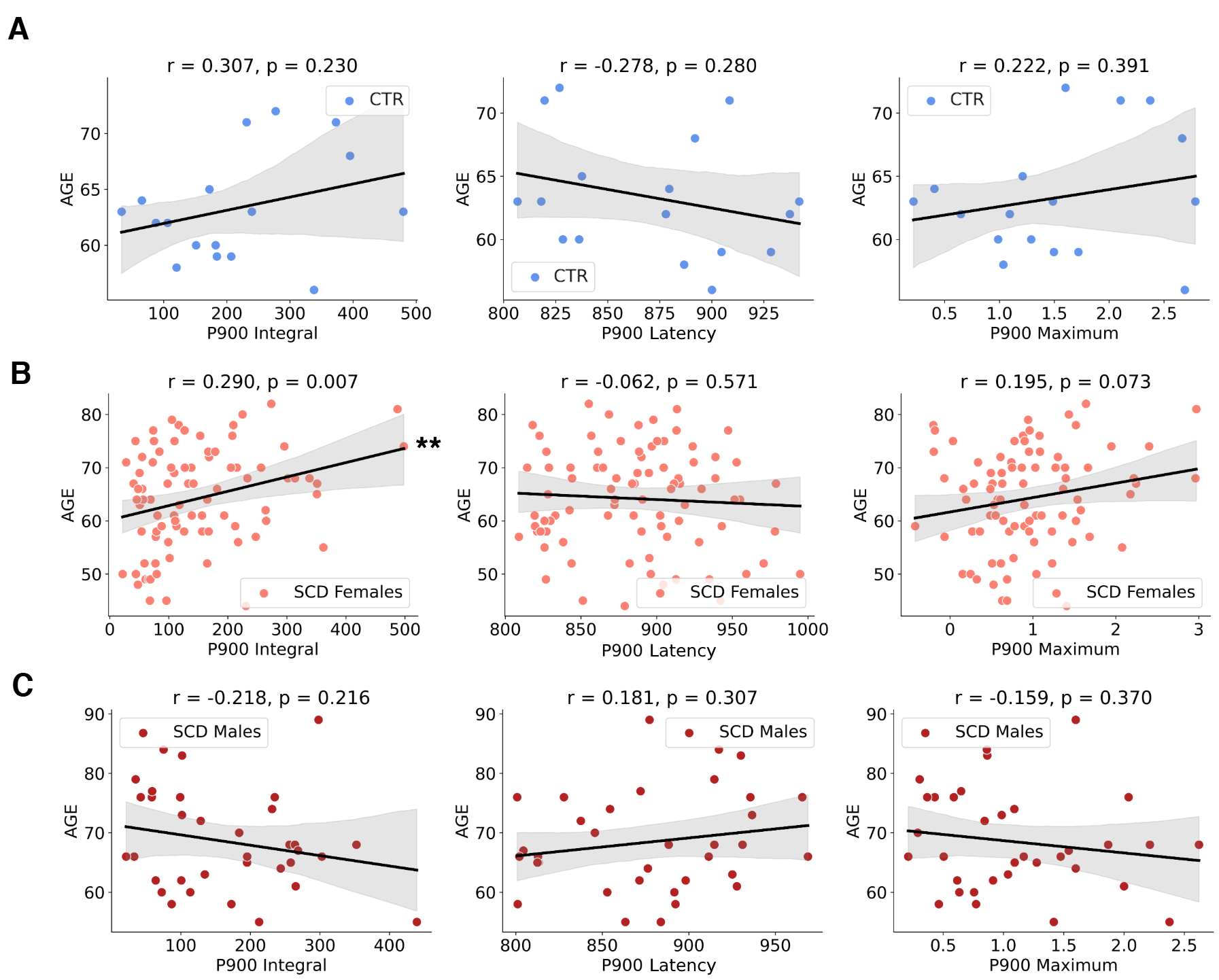


**Supplementary Figure 4: SCD Females and SCD Males participants present different evolution of the P900 component with age. (A):** Correlation between P900 integral values (left), latency values (middle) and maximum amplitude values (right) with age for the CTR group. Shaded area represents 2.5%-97.5% confidence interval. **(B):** Correlation between P900 integral values (left), latency values (middle) and maximum amplitude values (right) with age for the SCD Females group. Notation is the same as in (A). **(C):** Correlation between P900 integral values (left), latency values (middle) and maximum amplitude values (right) with age for the SCD Males group. Notation is the same as in (A). ** stands for p<0.01.


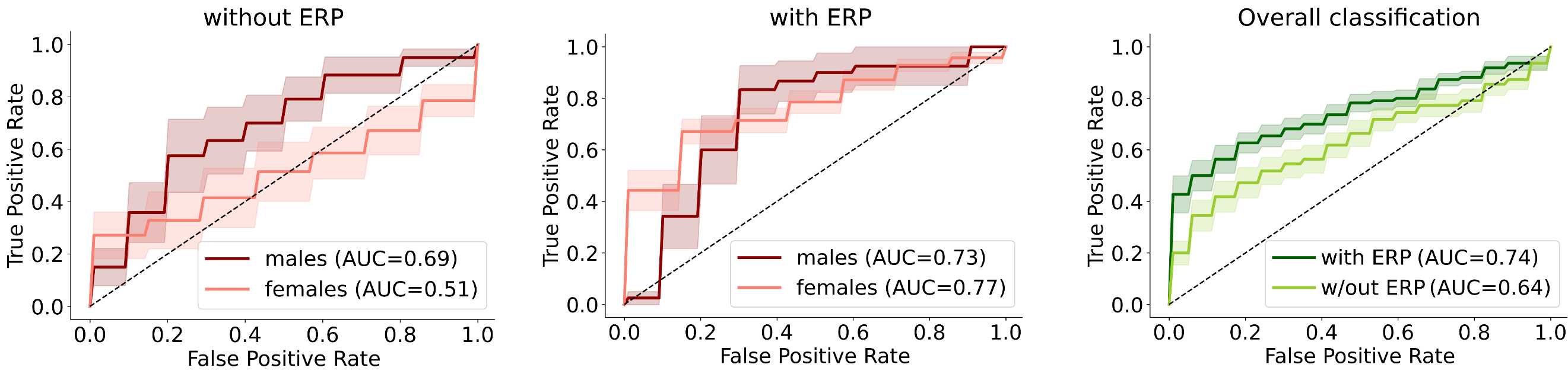


**Supplementary Figure 5: Classification results after balanced resampling. (A):** ROC curves for the sex-specific classifications between CTR and SCD participants made without ERP features. SCD Males and SCD Females are reported in dark red and salmon, respectively. **(B):** ROC curves for the sex-specific classifications between CTR and SCD participants made including ERP features. Notation is the same as in (A). **(C):** ROC curves for the overall (whole dataset) classifications between CTR and SCD participants, comparing results with and without ERP features. Results obtained using ERP features are reported in dark green, while results without ERP features are reported in yellow green.

**References**

1. Mazzeo S, Lassi M, Padiglioni S, et al. PRedicting the EVolution of SubjectIvE Cognitive Decline to Alzheimer’s Disease With machine learning: the PREVIEW study protocol. *BMC Neurol*. 2023;23(1):300. doi:10.1186/s12883-023-03347-8

2. Levine DA, Gross AL, Briceño EM, et al. Sex Differences in Cognitive Decline Among US Adults. *JAMA Netw Open*. 2021;4(2):e210169. doi:10.1001/jamanetworkopen.2021.0169

3. Vergani AA, Mazzeo S, Moschini V, et al. Event-related potential markers of subjective cognitive decline and mild cognitive impairment during a sustained visuo-attentive task. *NeuroImage Clin*. 2025;45:103760. doi:10.1016/j.nicl.2025.103760

4. Mazure CM, Swendsen J. Sex differences in Alzheimer’s disease and other dementias. *Lancet Neurol*. 2016;15(5):451-452. doi:10.1016/S1474-4422(16)00067-3

5. Doan DNT, Ku B, Choi J, et al. Predicting Dementia With Prefrontal Electroencephalography and Event-Related Potential. *Front Aging Neurosci*. 2021;13. doi:10.3389/fnagi.2021.659817

6. Albert MS, DeKosky ST, Dickson D, et al. The diagnosis of mild cognitive impairment due to Alzheimer’s disease: recommendations from the National Institute on Aging‐Alzheimer’s Association workgroups on diagnostic guidelines for Alzheimer’s disease. *Alzheimers Dement*. 2011;7(3):270-279.

7. Danjou P, Viardot G, Maurice D, et al. Electrophysiological assessment methodology of sensory processing dysfunction in schizophrenia and dementia of the Alzheimer type. *Neurosci Biobehav Rev*. 2019;97:70-84. doi:10.1016/j.neubiorev.2018.09.004

8. Gluhm S, Goldstein J, Loc K, Colt A, Van Liew C, Corey-Bloom J. Cognitive Performance on the Mini-Mental State Examination and the Montreal Cognitive Assessment Across the Healthy Adult Lifespan. *Cogn Behav Neurol Off J Soc Behav Cogn Neurol*. 2013;26(1):1-5. doi:10.1097/WNN.0b013e31828b7d26

9. Colombo L, Brivio C, Benaglio I, Siri S, Cappa SF. Alzheimer Patients’ Ability to Read Words with Irregular Stress. *Cortex*. 2000;36(5):703-714. doi:10.1016/S0010-9452(08)70547-1

10. Nelson H. National Adult Reading Test (NART): For the Assessment of Premorbid Intelligence in Patients with Dementia: Test Manual. *No Title*. Published online 1982. Accessed February 29, 2024. https://cir.nii.ac.jp/crid/1370846644342763143

11. Bessi V. *PRedicting the EVolution of SubjectIvE Cognitive Decline to Alzheimer’s Disease With Machine Learning*. clinicaltrials.gov; 2022. Accessed January 1, 2024. https://clinicaltrials.gov/study/NCT05569083
